# BMC Biochemistry reviewer acknowledgement, 2014

**DOI:** 10.1186/s12858-015-0035-8

**Published:** 2015-02-17

**Authors:** Guangde Tu

**Affiliations:** Room 1006-1007 Financial Plaza, No. 333 Jiujiang Road, Huangpu District Shanghai, 200001 China

## Abstract

The editors of BMC Biochemistry would like to thank all our reviewers who have contributed their time to the journal in Volume 15 (2014).

## Reviewer acknowledgement

**Ana I. Azuaga**

Spain

**Nafisa Balasinor**

India

**David Bentley**

USA

**Tadayoshi Bessho**

USA

**Volker Briken**

USA

**Ana Busturia**

Spain

**Francisco Campos**

Brazil

**Guifang Cao**

China

**MI-LA Cho**

South Korea

**Mark Chong**

Australia

**Jane Elizabeth Collins**

United Kingdom

**Yu-Sheng Cong**

China

**Kalavathi Dasuri**

USA

**Laurent Desaubry**

France

**Maria Grazia Esposito**

Italy

**Daniela Fietz**

Germany

**Theodore Fotsis**

Greece

**Nicole Francis**

Canada

**Octavio Franco**

Brazil

**Christian Frezza**

United Kingdom

**Suthat Fucharoen**

Thailand

**Ramesh Ganju**

USA

**Jan Gettemans**

Belgium

**Celia Goulding**

USA

**Matt Guille**

United Kingdom

**Hervé Guillou**

France

**Marina Guizzetti**

USA

**Bassem Hassan**

Belgium

**Harald Herrmann**

Germany

**Michal Hetman**

USA

**Jim Horn**

USA

**Jianghui Hou**

USA

**Thomas Hurley**

USA

**Ibtessam Hussein**

Saudi Arabia

**Kazumi Ishidoh**

Japan

**Atsuo Iwasawa**

Japan

**Marek Jankoiwski**

Canada

**Xinhua Ji**

USA

**Anne Katherine Jones**

USA

**Bavesh Davandra Kana**

South Africa

**Paul King**

USA

**Essam Kotb**

Egypt

**Adam Krieg**

USA

**Isabelle Landrieu**

France

**Konstantinos Lefkimmiatis**

United Kingdom

**Christina Leslie**

USA

**Jie Li**

USA

**Stefan Lichtenthaler**

Germany

**Jing-Jer Lin**

Taiwan

**Peter Lindblad**

Sweden

**Dan Lindholm**

Finland

**ZuFu Lu**

Australia

**Erich Mackow**

USA

**Lee Macomber**

USA

**Sandro Marana**

Brazil

**Neil McDonald**

United Kingdom

**John McLenithan**

USA

**Rod Merrill**

Canada

**Joris Messens**

Belgium

**John Mieyal**

USA

**Isabelle Mus-Veteau**

France

**Masato Nakai**

Japan

**Fengyun Ni**

USA

**Ken Okazaki**

Japan

**Angela Pennacchio**

Italy

**Giulia Piaggio**

Italy

**Miles Pufall**

USA

**Pradman Qasba**

USA

**Jose Luis R. Arrondo**

Spain

**Arumugam Rajavelu**

India

**Francois Robert**

Canada

**Christoph Romanin**

Austria

**George Russev**

Bulgaria

**Avneesh Saini**

USA

**Roger Sandwick**

USA

**Neelam Sangwan**

India

**Devanand Sarkar**

USA

**Jitendra Saxena**

India

**Gerd Schmitz**

Germany

**Claudia Scholl**

Germany

**Tobias Schuerholz**

Germany

**Benjamin Schulz**

Australia

**Dominik Schwudke**

Germany

**Jose Segovia**

Mexico

**Olli Silvennoinen**

Finland

**Alexander Skurat**

USA

**Guanfang Su**

China

**Shuo Sun**

USA

**Avadhesha Surolia**

India

**Sanjay Swarup**

Singapore

**Liskin Swint-Kruse**

USA

**Daniel Taillandier**

France

**Yuichiro Takanami**

Japan

**Tien-Chye Tan**

Sweden

**Winston Thompson**

USA

**Nguyen Trong Tue**

Viet Nam

**Kouhei Tsumoto**

Japan

**Takafumi Uchida**

Japan

**John Ussher**

Canada

**Martin van der Laan**

Germany

**Cornelis Van Noorden**

Netherlands

**Joseph Vasselli**

USA

**Roberto Vazquez-Padron**

USA

**Jan Willem Voncken**

Netherlands

**Geerten W. Vuister**

United Kingdom

**Chong Wang**

China

**Oliver Weichenrieder**

Germany

**Catherine Winder**

United Kingdom

**Dieter Wolf**

USA

**Kinnosuke Yahiro**

Japan

**Zhaogang Yang**

USA

**Lennart Zabeau**

Belgium

**Jianye Zang**

China

**Haili Zhang**

USA

**Guoqing Zhang**

USA

